# Bowel Obstruction Caused by Broad Ligament Hernia Successfully Repaired by Laparoscopy in an Elderly Patient: A Case Report

**DOI:** 10.7759/cureus.23237

**Published:** 2022-03-16

**Authors:** Mohammed Marzouk, Ali Al Abdulsalam, Aliaa Soliman

**Affiliations:** 1 Department of General Surgery, Al-Adan Hospital, Kuwait, KWT; 2 Department of Radiology, Al-Adan Hospital, Kuwait, KWT

**Keywords:** case report, surgery, laparoscopy, small bowel obstruction, internal hernia, broad ligament

## Abstract

The most common causes of small bowel obstruction (SBO) are adhesions, Crohn’s disease, neoplasms, and hernias. Internal hernias are rare, and they occur when the small bowel herniates through a defect in the abdominal cavity. The occurrence of internal hernias due to a broad ligament defect is very rare and accounts for 4%-7% of cases of internal hernia.

We present a case of a 71-year-old female who was previously healthy with no significant past medical or surgical history and who presented with symptoms of small bowel obstruction. Imaging with X-ray and computed tomography (CT) confirmed the diagnosis, but not the etiology. A decision was made to perform a laparoscopy to manage the obstruction, which revealed a healthy small bowel loop that herniated through a defect in the right broad ligament.

Acute abdominal pain due to intestinal obstruction is a relatively common surgical emergency. Internal hernias are the consequence of the herniation of a bowel loop, most commonly the small bowel, through a peritoneal or mesenteric defect into a compartment in the abdominal and pelvic cavity, and they have a high mortality rate than can be higher than 50%. CT imaging is very useful in the diagnosis of internal hernias, although it may not always reveal the etiology. To facilitate wider recognition, broad ligament hernia should be in the differential diagnosis of internal hernias evident in the pelvis on CT imaging.

Early recognition of small bowel obstruction caused by broad ligament internal hernia allows for prompt surgical management and vastly facilitates postoperative recovery. Although most surgeons opt for a laparotomy approach to manage such cases, a laparoscopic approach is feasible.

## Introduction

The most common causes of small bowel obstruction (SBO) are adhesions, Crohn’s disease, neoplasms, and hernias [1]. The occurrence of internal hernias is quite rare and accounts for 0.5%-4.1% of cases of intestinal obstruction [2]. Internal hernias arise when a portion of the small bowel herniates through a peritoneal or mesenteric defect into a compartment in the abdominal and pelvic cavity [3]. The occurrence of internal hernias due to a broad ligament defect is very rare and accounts for 4%-7% of cases of internal hernia [2,3]. We present an unusual case of a 71-year-old female who presented with symptoms of SBO through a defect in the right broad ligament that was successfully repaired by laparoscopic surgery. The case is presented in line with the Surgical CAse REport (SCARE) criteria [4].

## Case presentation

**Figure 1:**
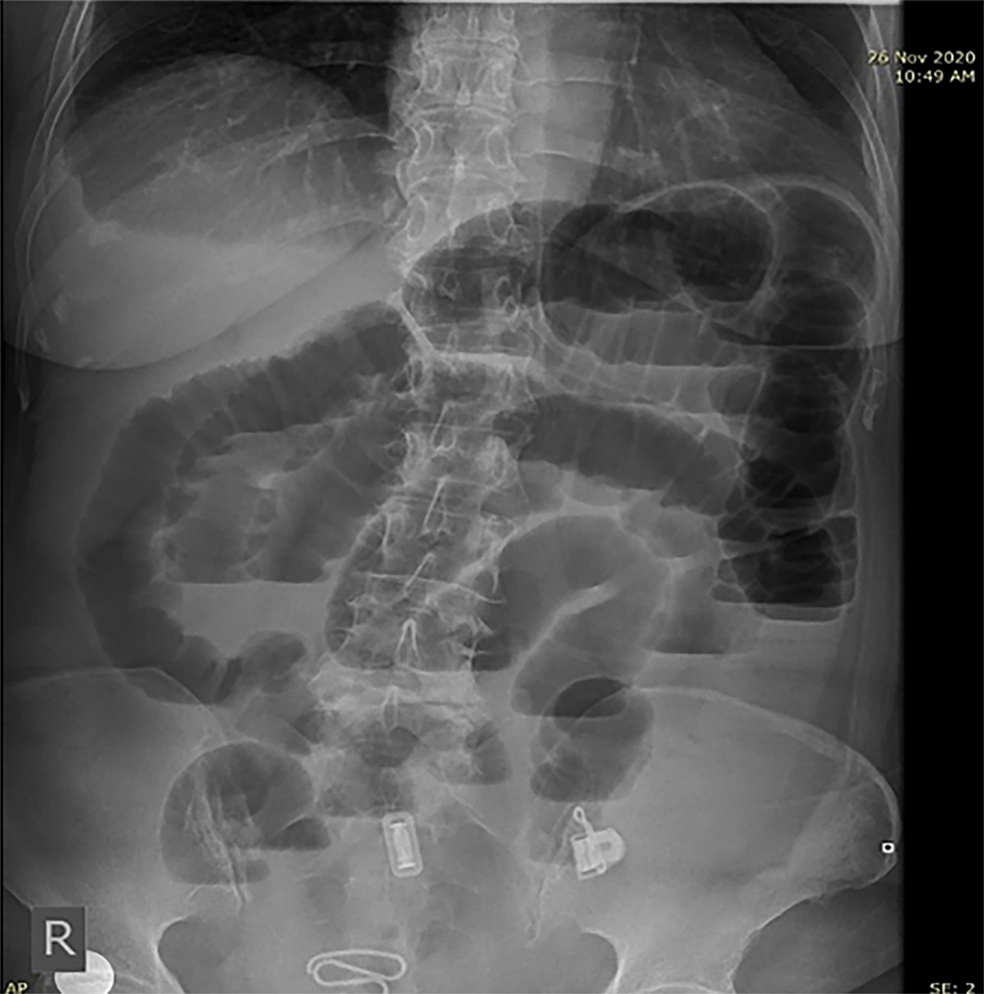
Erect abdominal radiograph showing dilated small bowel loops with multiple air-fluid levels suggestive of small bowel obstruction.

A previously healthy 71-year-old female self-presented to our general hospital with a few days history of abdominal pain that was colicky in nature, constipation, nausea, and vomiting. There was a gradual worsening of her symptoms, and oral intake became intolerable. She has no family history of inheritable conditions or drug allergies. There was no past medical or surgical history of note. On examination, she was apyrexial and not tachycardic. There was moderate abdominal distension, but no local tenderness to palpation. Laboratory investigations revealed a slight increase in the white blood cell (WBC) count of 15 × 10^9^/L. An erect abdominal radiograph showed distended small bowel loops with multiple air-fluid levels (Figure 1).

**Figure 2:**
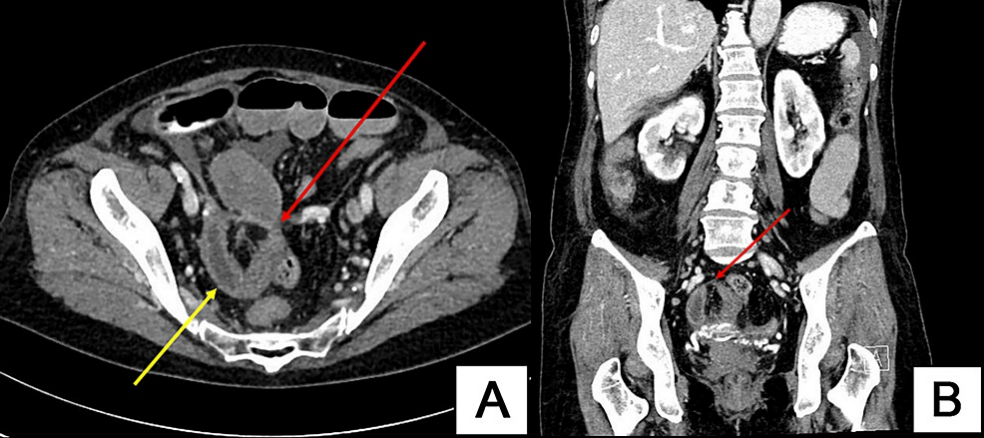
(A) Axial CT pelvis showing U-shaped closed small bowel loop (yellow arrow) and point of obstruction (red arrow). (B) Coronal CT showing closed loop neck (red arrow).

**Figure 3:**
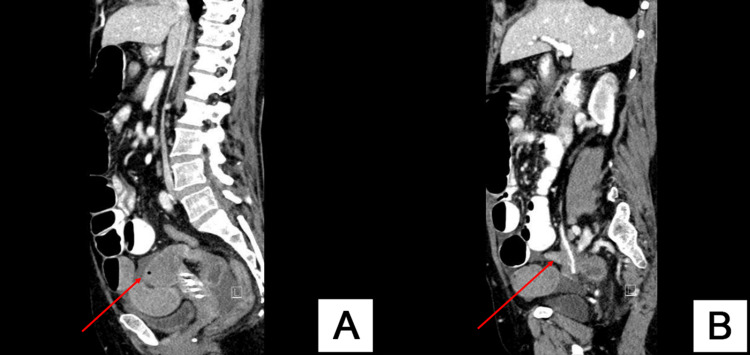
(A) Sagittal CT showing afferent dilated small bowel loop (red arrow). (B) Sagittal CT showing efferent collapsed small bowel loop (red arrow).

Abdominal ultrasound was done outside the hospital and revealed a distended stomach and bowels. Abdominopelvic computed tomography (CT) showed dilated small bowel loops with a closed loop zone of transition suggestive of mechanical SBO secondary to internal hernia (Figures 2, 3).

**Figure 4:**
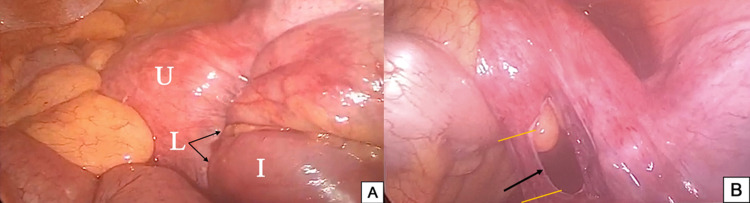
(A) Intraoperative photograph. (B) Intraoperative photograph showing a defect in the right broad ligament (black arrow) where the terminal ileal loop was pulled out from (yellow lines: two surgical clips were applied, and a small segment the broad ligament in between was excised). U: uterus, L: broad ligament, I: ileal loop

A decision for diagnostic laparoscopy was made to discern the etiology of the mechanical obstruction. The laparoscopy showed a small bowel loop herniating through the right broad ligament (Figure 4A). The healthy loop of the small bowel was pulled out of the defect in the right broad ligament. Two surgical clips were applied as seen in Figure 4B, and a 2-cm small segment of the broad ligament between the clips was excised to prevent a recurrence. There were no complications postoperatively, oral intake was initiated the following day, and the patient was discharged two days thereafter.

## Discussion

We present a case of a previously healthy 71-year-old female with no significant past medical or surgical history who self-presented with symptoms suggestive of SBO. With the aid of imaging modalities such as erect abdominal radiograph and CT scan of the abdomen with intravenous (IV) contrast, a provisional diagnosis of internal hernia was made. Upon laparoscopic surgery, it was found that the internal hernia was caused by a defect in the right broad ligament.

Acute abdominal pain due to intestinal obstruction is a relatively common surgical emergency diagnosed in 15% of emergency department presentations [5]. The most common cause of SBO is adhesions following abdominal surgery [1]. Internal hernias are the consequence of the herniation of a bowel loop, most commonly the small bowel, through a peritoneal or mesenteric defect into a compartment in the abdominal and pelvic cavity, and they have a high mortality rate than can be higher than 50% [6,7]. The numerous types of internal hernias include paraduodenal (53%), pericecal (13%), foramen of Winslow (8%), sigmoid (6%), and transmesenteric (8%) [3]. The least common of all internal hernias are broad ligament hernias, accounting for 4%-7% of cases of internal hernia [2].

In 1984, a classification that consists of three types of broad ligament hernias according to the degree of the defect was proposed by Hunt. The most common type is fenestra, which involves the two peritoneal layers of the broad ligament as seen in our case. The second type is the pouch type; it occurs when only one of the two layers is involved, which results in the entrapment of visceral structures in the parametrial tissue. The last type is the hernia sac type, in which the herniated bowel is lined by a double layer of peritoneum resulting in a true internal hernia [6].

A new classification by Cilley et al. was introduced according to the anatomical location of the defect and has three types: a defect posterior to the round ligament, a defect superior to the broad ligament, and a defect between the round ligament and the remaining broad ligament, through the mesoligametum teres [8]. Our case was the first type in accordance with Cilley et al.’s classification.

It is not necessary to reach a diagnosis of broad ligament hernia before surgery owing to the fact that complete or closed loop bowel obstruction requires surgical intervention. CT has precedence in the diagnosis of SBO and investigating its etiology. Findings of broad ligament herniation on CT include the presence of a transition zone in the pelvis, lateral herniation of dilated small bowel loops to the pelvic cavity, and an increased distance between the uterus and one of the ovaries [9,10].

In our case, we did not reach a diagnosis of broad ligament hernia prior to surgery and only anticipated that the etiology was pelvic in origin based on CT imaging. Most surgeons address SBO by performing a laparotomy [11]. Although the laparoscopic approach is becoming more common, most surgeons are hesitant due to the fear of causing an iatrogenic intestinal injury and the tight space due to distended bowel loops [12]. According to current evidence, however, laparoscopy is sufficient in the management of SBO caused by internal hernia of the broad ligament and vastly facilitates postoperative recovery [10].

## Conclusions

The differential diagnosis of internal hernia through a defect in the broad ligament, although rare, should be considered when a female presents with intestinal obstruction. Early recognition of SBO caused by broad ligament internal hernia allows for prompt surgical management and considerably facilitates postoperative recovery. CT plays an important role in identifying SBO and may or may not indicate the etiology. Although most surgeons opt for a laparotomy approach to manage such cases, a laparoscopic approach is feasible.
